# Evaluation of the Cross-Protective Efficacy of a Chimeric PRRSV Vaccine against Two Genetically Diverse PRRSV2 Field Strains in a Reproductive Model

**DOI:** 10.3390/vaccines9111258

**Published:** 2021-10-31

**Authors:** Chang-Gi Jeong, Amina Khatun, Salik Nazki, Seung-Chai Kim, Yun-Hee Noh, Sang-Chul Kang, Dong-Uk Lee, Myeon-Sik Yang, Nadeem Shabir, In-Joong Yoon, Bumseok Kim, Won-Il Kim

**Affiliations:** 1 College of Veterinary Medicine, Jeonbuk National University, Iksan 54596, Korea; jcg0102@gmail.com (C.-G.J.); amina.vet.sau.bd@gmail.com (A.K.); saliknazki@gmail.com (S.N.); leesor2@jbnu.ac.kr (S.-C.K.); 111@jbnu.ac.kr (M.-S.Y.); drnadurose@gmail.com (N.S.); bskims@jbnu.ac.kr (B.K.); 2 Department of Pathology, Faculty of Animal Science and Veterinary Medicine, Sher-e-Bangla Agricultural University, Dhaka 1207, Bangladesh; 3 The Pirbright Institute, Pirbright GU24 0NF, UK; 4 ChoongAng Vaccine Laboratory, Daejeon 34055, Korea; sdocter@cavac.co.kr (Y.-H.N.); showsh233@gmail.com (D.-U.L.); jyoon@cavac.co.kr (I.-J.Y.); 5 Animal Clinical Evaluation Center, Optipharm Inc., Cheongju-si 28158, Korea; supervet@naver.com; 6 Division of Animal Biotechnology, Faculty of Veterinary Sciences and Animal Husbandry, Sher-e-Kashmir University of Agricultural Sciences and Technology of Kashmir, Srinagar 190006, India

**Keywords:** porcine reproductive and respiratory syndrome, PRRSV, reproductive model, reproductive failure, PRRS vaccine, chimeric vaccine

## Abstract

Despite the routine use of porcine reproductive and respiratory syndrome (PRRS)-modified live vaccines, serious concerns are currently being raised due to their quick reversion to virulence and limited cross-protection against divergent PRRS virus (PRRSV) strains circulating in the field. Therefore, a PRRS chimeric vaccine (JB1) was produced using a DNA-launched infectious clone by replacing open reading frames (ORFs) 3–6 with those from a mixture of two genetically different PRRSV2 strains (K07–2273 and K08–1054) and ORF1a with that from a mutation-resistant PRRSV strain (RVRp22) exhibiting an attenuated phenotype. To evaluate the safety and cross-protective efficacy of JB1 in a reproductive model, eight PRRS-negative pregnant sows were purchased and divided into four groups. Four sows in two of the groups were vaccinated with JB1, and the other 4 sows were untreated at gestational day 60. At gestational day 93, one vaccinated group and one nonvaccinated group each were challenged with either K07–2273 or K08–1054. All of the sows aborted or delivered until gestation day 115 (24 days post challenge), and the newborn piglets were observed up to the 28th day after birth, which was the end of the experiment. Overall, pregnant sows of the JB1-vaccinated groups showed no meaningful viremia after vaccination and significant reductions in viremia with K07–2273 and K08–1054, exhibiting significantly higher levels of serum virus-neutralizing antibodies than non-vaccinated sows. Moreover, the JB1-vaccinated groups did not exhibit any abortion due to vaccination and showed improved piglet viability and birth weight. The piglets from JB1-vaccinated sows displayed lower viral concentrations in serum and fewer lung lesions compared with those of the piglets from the nonvaccinated sows. Therefore, JB1 is a safe and effective vaccine candidate that confers simultaneous protection against two genetically different PRRSV strains.

## 1. Introduction

Porcine reproductive and respiratory syndrome (PRRS) has been the most challenging threat to the swine industry worldwide for over two decades. PRRS causes economic losses, with an estimated annual loss of approximately $664 million in the USA alone. Over $300 million of this loss is due to reproductive failure associated with the PRRS virus (PRRSV) [1,2,3,4]. Reproductive failure is characterized by abortion, mummified fetuses, weak birth and stillbirth, postweaning pneumonia, increased mortality, and growth retardation of young pigs [3,5]. The causative agent, PRRSV, is a single-stranded positive-sense RNA virus (~15 kb) that is classified to the *Betaaarterivirus* by the International Committee on Taxonomy of Viruses (ICTV), belonging to the order *Nidovirales*, the *Arteriviridae* family [6,7,8,9]. The PRRSV genome encodes at least 10 open reading frames (ORFs) consisting of ORF1a, ORF1b, ORF2a, ORF2b, ORF3, ORF4, ORF5a, ORF5, ORF6, and ORF7 [10]. ORF1a and ORF1b encode nonstructural proteins (nsps) that are associated with virus replication [11]. ORF2a to ORF4 encode minor structural proteins (GP2, E, GP3 and GP4), and small amounts of structural proteins are encoded by ORF5a. The major structural proteins GP5, matrix (M) and nucleocapsid (N) are encoded by ORF5, 6 and 7, respectively [12]. GP5 has been considered an important protein for targeting by virus-neutralizing (VN) antibodies [13,14,15,16]. In addition, the GP3, GP4, and M proteins have also been reported to play roles in inducing the production of VN antibodies [16,17,18].

Based on sequence analysis by the ICTV, the two genotypes of PRRSV, PRRSV1 and PRRSV2, are classified into two distinct viral species as *Betaaarterivirus suid* 1 and *Betaaarterivirus* 2 [9]. High levels of genetic variability exist within PRRSVs, which is caused by mutations and recombination among PRRSV strains [19]. Based on the ORF5 sequence, PRRSV2 was classified into ninedistinct lineages [20]. In Korea, there are new Korean PRRSV lineages (Kor A, Kor B, and Kor C) that are unlike the existing lineages [20,21,22]. According to a recent report, the majority of Korean PRRSV2 isolates belong to lineage 5 (L5) and Korean lineages. Kor A was first reported in 2010 [23], but Kor B and C were first classified in 2014 [22].

The genetic diversity of PRRSV results in suboptimal cross-protection among different PRRSV strains and is an impediment to vaccine development [24]. PRRS-modified live vaccines (MLVs) have been used to control PRRSV, providing better homologous protection than killed PRRSV vaccines or recombinant vaccines [25]. However, a major problem in the use of PRRS MLVs is their limited cross-protection [26,27,28]. Additionally, the use of PRRS MLVs has serious safety issues due to quick reversion to virulence of the vaccine strains during serial passages in pigs [29,30,31].

To overcome the major problem of current PRRS MLVs, which lack cross-protection activity, various PRRSV infectious clones have been constructed to improve the cross-protection range. A previous study demonstrated that chimeric PRRSV, which contained mixed structural genes from two different strains, was able to provide cross-protection against donor strains [16]. In another previous study, it was observed that a chimeric PRRSV (K418) consisting of the structural gene of the LMY strain in the FL12 backbone produced cross-protection in vitro [32]. Subsequently, the same research team created deglycosylated K418 (K418DM), which was proven to be effective and safe under experimental and field conditions [33]. A recent study also reported that PRRSV chimeras that were modified using DNA shuffling methods with six heterologous PRRSV strains exhibited improved cross-protective efficacy against heterologous PRRSV strains [34]. Together, these studies implied that chimeric PRRSV consisting of mixed structural genes is an effective strategy to broaden cross-protection against various PRRSV strains. Similarly, in our previous study [28], A PRRS chimeric virus (CV) was constructed by an FL12-based DNA-launched infectious clone, in which ORFs 3–4 and ORFs 5–6 were swapped with those of two different PRRSV2 isolates, K08–1054 (L5) and K07–2273 (KorC), respectively. CV was evaluated for cross-protective efficacy against two genetically distinct PRRSV2 strains (K07–2273 and K08–1054) using a young pig model. The CV-vaccinated group displayed the highest average daily weight gain (ADWG) among the experimental groups. In addition, 50% of CV-vaccinated pigs showed a serum virus neutralization (SVN) titer of ≥1:32 against K07–2273 and K08–1054 and exhibited a significant reduction in viral loads in comparison with those of pigs in the mock groups at 42 days post vaccination (dpv). Increased levels of tumor necrosis factor-alpha (TNF-α), interferon-gamma (IFN-γ), and interleukin-12 (IL-12) and decreased IL-10 levels were detected in peripheral blood mononuclear cells (PBMCs), indicating that CV induced a cell-mediated immune response and might be associated with lower viral loads in serum.

Another major challenge to PRRS vaccinology is that PRRS MLVs can quickly revert to virulence, resulting in safety concerns [25]. Several studies have reported that mutation-resistant viruses reemerged via the presence of sublethal concentrations of antiviral components, and these viruses were more genetically stable than their parental viruses [35,36,37,38]. Our previous study reported that mutagen-resistant mutants emerged at 22 passages (RVRp22) when treated with ≤0.2 mM ribavirin (antiviral component). RVRp22 showed a significantly lower mutation rate in nsp2 and ORF5 than VR2332 after 10 passages in MARC145 cells [24]. Subsequently, in a previous study [39], RVRp22 was evaluated in terms of genetic and phenotypic stability in vivo. Seven unique amino acid mutations were found in ORF1a of RVRp22 (465S, 788L, 1019E, 1186V, 1248H, and 1375F in nsp2 and 2400T in nsp7), which might be responsible for viral genetic stability, attenuation, and virulence in pigs. Moreover, the attenuation phenotype of RVRp22 was maintained during sequential passages in pigs. In the present study, a new chimeric vaccine (JB1) was constructed by replacing ORF1a with RVRp22 using a DNA-launched infectious clone of CV to improve genetic stability and cross-protection ranges. Here, the vaccine was evaluated for its safety and efficacy in a reproductive model. To our knowledge, this is the first experiment to evaluate a chimeric vaccine in a reproductive model.

## 2. Materials and Methods

### 2.1. PRRSV Isolates

The Korean PRRSV2 strains K07–2273 (GenBank accession number: JQ656251; Kor C) and K08–1054 (GenBank accession number: JQ656266; L5) were used in this study. These PRRSV2 isolates were propagated in MARC-145 (African green monkey) cells. MARC-145 cells used for virus culture were maintained in RPMI-1640 medium supplemented with heat-inactivated 10% fetal bovine serum (FBS; Invitrogen, Carlsbad, CA, USA), 2 mM L-glutamine, and 100X antibiotic-antimycotic solution [Anti-anti, Invitrogen; 1X solution contains 100 IU/mL penicillin and 100 μg/mL Fungizone^®^ (amphotericin B)] at 37 °C in a humidified 5% CO_2_ atmosphere.

### 2.2. Construction of Chimeric PRRSV (JB1)

**Figure 1:**
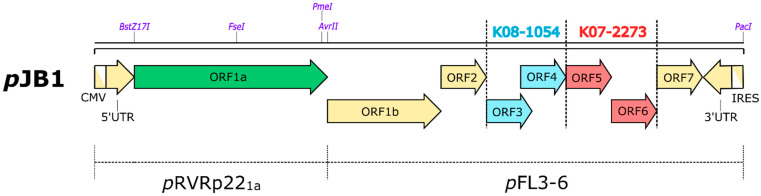
Graphical representation of the genomic construct for chimeric infectious clone *p*JB1 (*p*RVRp22–1aK3–6). The restriction sites used for cloning are listed above the construct. CMV: human cytomegalovirus; IRES: internal ribosomal entry site; *BstZ17I*, *FseI*, *AvrII*, *PmeI*, *PacI*: restriction sites.

The chimeric infectious clone *p*JB1 (*p*RVRp22–1aK3–6) was constructed by replacing ORF1a from RVRp22 (a highly genetically stable, ribavirin-resistant attenuated PRRSV) into *p*FL3–6 (a chimeric infectious clone of CV) [28,39]. For that purpose, a chimeric infectious clone, *p*RVRp22_1a_, was constructed according to the concepts of previous studies [40,41]. ORF1a from the RVRp22 [39] genome was inserted into a modified VR2332-based infectious clone (*p*VR2332/a2) as the backbone using a reverse genetic approach [42]. Shortly, the shuttle vector sRVRp22_1a_ (sRVRp22_1a1_ + sRVRp22_1a2_) [containing the ORF1a (divided into two parts, ORF1a1 and ORF1a2) region from the RVRp22 genome] was constructed according to a previous study [41] to produce RVRp22-based ORF1a (nonstructural gene-containing shuttle vector). For that, viral RNA was extracted from the RVRp22 genome with a commercial kit (Ribo_spin vRDTM, GeneAll, Seoul, Korea) and amplified with primers (Table 1) designed for each respective region [42] using a high-fidelity one-step RT-PCR kit (SuperScript^®^ One-Step RT-PCR for Long Template, Invitrogen, Carlsbad, CA, USA) according to the manufacturer’s guidelines. Then, the amplified PCR products were gel-purified using a commercial kit (Wizard^®^ SV Gel and PCR Clean-Up System, Promega) and cloned into the pGEM^®^-T Easy vector system (Promega Corporations, Madison, WI, USA) using *SphI* and *SpeI* to produce the chimeric shuttle vector sRVRp22_1a_ (sRVRp22_1a1_ + sRVRp22_1a2_). Before being used for assembly of the full-length chimeric infectious clone, the individual subclones for each part of the shuttle vectors were sequenced (Macrogen, Inc., South Korea) to confirm the sequences. All the primers used in construction and sequencing are listed in Table 1. Then, three-point ligation (*BstZ17I*+*FseI* from sRVRp22_1a1_, *FseI*+*AvrII* from sRVRp22_1a2_, and *AvrII*+*BstZ17I* from the backbone infectious clone, pVR2332/a2) was conducted to construct the chimeric infectious clone *p*RVRp22_1a_. Finally, the new chimeric infectious clone *p*JB1 (*p*RVRp22–1aK3–6) was constructed by swapping the two chimeric infectious clones *p*RVRp22_1a_ and *p*FL3–6 [28] using two common enzymes *PmeI* and *PacI* (Figure 1).

The chimeric virus (JB1) was rescued in 24-well cell culture plates by transfecting the chimeric infectious cDNA clone (*p*JB1) into MARC-145 cells using the electroporation method described in previous studies [18,28,43]. The Rescued JB1 was then propagated sequentially three times from a 24-well cell culture plate to in a 25 cm^2^ to in a 75 cm^2^ cell culture flask (BD, Falcon) to obtain higher amounts of virus. After 3 freeze thaws, the JB1 cultured third time in the 75 cm^2^ cell culture flask was collected, centrifuged, and stored at −80 °C after titration until use. The sequence of the chimeric virus was confirmed again by sequencing, and the full-length JB1 sequences were deposited in GenBank (accession number: MZ416787).

### 2.3. Animal Study

**Figure 2:**
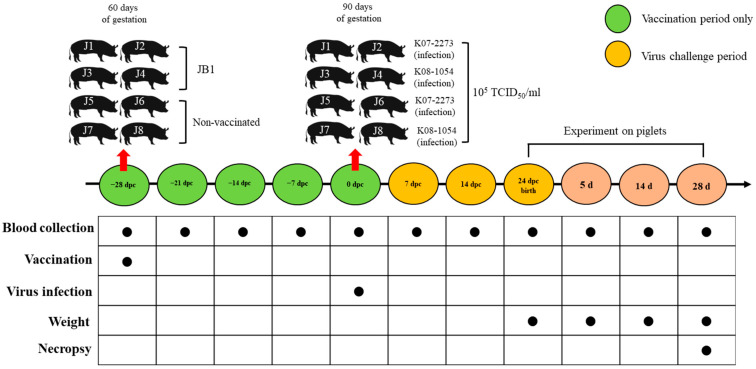
Study design. Pregnant sows were intramuscularly vaccinated with JB1 at 10^5^ TCID_50_/mL at 60 days of gestation and inoculated with field isolates intranasally at 10^5^ TCID_50_/mL at 28 dpv (0 dpc). Blood collection was conducted at specific time points, and weighing was performed for piglets only.

The design of the present study is shown in Figure 2. Eight seronegative pregnant sows were purchased from a PRRSV-free farm. Pregnant sows were randomly housed and divided into 4 groups. Pregnant sows were numbered J1 to J8. The J1–J4 pregnant sows were intramuscularly vaccinated (60 days of gestation) with JB1 at 10^5^ 50% tissue culture infective dose (TCID_50_)/mL, and the J5–J8 pregnant sows were kept as nonvaccinated (NV) groups. At 28 days post vaccination [dpv; 0 days post challenge (dpc)], J1–J2 and J3–J4 were intranasally inoculated with K07–2273 and K08–1054 at 10^5^ TCID_50_/mL, respectively, at 90 days of gestation. J5–J6 and J7–J8 were also intranasally inoculated with K07–2273 and K08–1054 at 10^5^ TCID_50_/mL as the challenged groups (NV/K07–2273 and NV/K08–1054) on the same day described above. On the date of birth, the survival of neonates was recorded.

Sera were collected from the sows at −28 (JB1 vaccination), −21, −14, −7, 0 (virus challenge), 7, 14, and 24 dpc for virological and serological assays. The piglets were weighed, and their sera were tested via the same assays at 0 (birth), 5, 14, and 28 days post birth (dpb). All piglets and sows were euthanized at 28 days post farrowing. Lung tissue samples were frozen at −80 °C until further experiments. For histopathology, the lung tissues were also placed in 10% neutral-buffered formalin. The animal experimental protocol was approved by the Jeonbuk National University Institutional Animal Care and Use Committee (approval number: 2016–0043).

### 2.4. Quantification of PRRSV RNA in Serum

Viral RNA was extracted from 100 µL of serum using a MagMAXTM Viral RNA Isolation Kit (Ambion, Applied Biosystems, Life Technologies, Inc., Carlsbad, CA, USA) according to the manufacturer’s instructions. The viral load in serum was measured using a real-time reverse transcription polymerase chain reaction (RT-PCR) employing a one-step qRT-PCR kit (Prime-Q PCV2, PRRSV Detection Kit, GeNet Bio, Inc., Daejeon, Korea) according to the manufacturer’s instructions with a 7500 Fast Real-time PCR system (Applied Biosystems, Foster City, CA, USA). To determine the PRRSV genome RNA copy number, a 1231-bp PRRSV2 ORF5 to ORF6 sequence (primers: F: 5′-GGTGGGCAACTGTTTTAGCCT-3′, R: 5′-GGCACAGCTGATTGACTGGC-3′) were cloned into the pGEM^®^-T Easy vector (Promega, Madison, WI, USA) according to the manufacturer’s instructions. Standard curves were generated from serial 10-fold dilutions of the plasmid constructs. The PRRSV genome absolute quantities were calculated by normalization to the standard curve.

### 2.5. Serology

PRRSV-specific antibodies (IgG) were detected in the serum using a commercially available ELISA kit (Bionote PRRS Ab 4.0, Hwasung, Korea) based on the nucleocapsid protein (N) according to the manufacturer’s instructions. The sample-to-positive (S/P) ratios of the samples were ≥0.4, which was considered PRRSV antibody-positive.

### 2.6. Serum Virus Neutralization Assay (SVN)

A fluorescent focus neutralization assay-based SVN assay was conducted to evaluate SVN antibody titers after vaccination and challenge. For the evaluation of cross-protective efficacy, antisera were tested against K07–2273 and K08–1054. The SVN assay was conducted as described previously [29]. The SVN titer of antiserum against K07–2273 and K08–1054 was expressed as the reciprocal of the highest dilution in which a 90% or higher reduction in the number of fluorescence focus-forming units (FFUs) was observed compared to that of the virus background titration.

### 2.7. Histopathological Evaluation

All lung tissue from sows underwent histopathological examination, while lung tissue from piglets was randomly selected from six piglets of each group and examined. Approximately 2 cm^3^ of sow and piglet samples were fixed in 10% phosphate-buffered formalin, routinely processed, and then embedded in paraffin. Tissue sections (4 µm) were prepared using a microtome (HM-340E, Thermo Fisher Scientific, Inc., Waltham, MA, USA). Sections were placed onto glass slides. Hematoxylin and eosin (H&E) staining was performed according to standard techniques. The microscopic lesions of the lung were given a score of 0–4 following a previous study [44]. Briefly, the scores assigned were as follows: 0, no lesion; 1, mild interstitial pneumonia; 2, moderate multifocal interstitial pneumonia; 3, moderate diffuse interstitial pneumonia; and 4, severe interstitial pneumonia.

### 2.8. Statistical Analysis

Two-way ANOVA with Tukey’s multiple comparison test was used to analyze the significance of variability within experimental groups for viremia and anti-PRRSV antibodies from sows and piglets. A *t*-test (Mann-Whitney test) was used to compare the weight of live neonates. Differences were considered statistically significant at *p* < 0.05. GraphPad Prism 7.00 (GraphPad Software, Inc., San Diego, CA, USA) was used to generate graphs, and statistical analysis was performed using SPSS Advanced Statistics 17.0 software (SPSS, Inc., Chicago, IL, USA).

## 3. Results

### 3.1. Quantification of Viral Load in Sow Samples 

**Figure 3:**
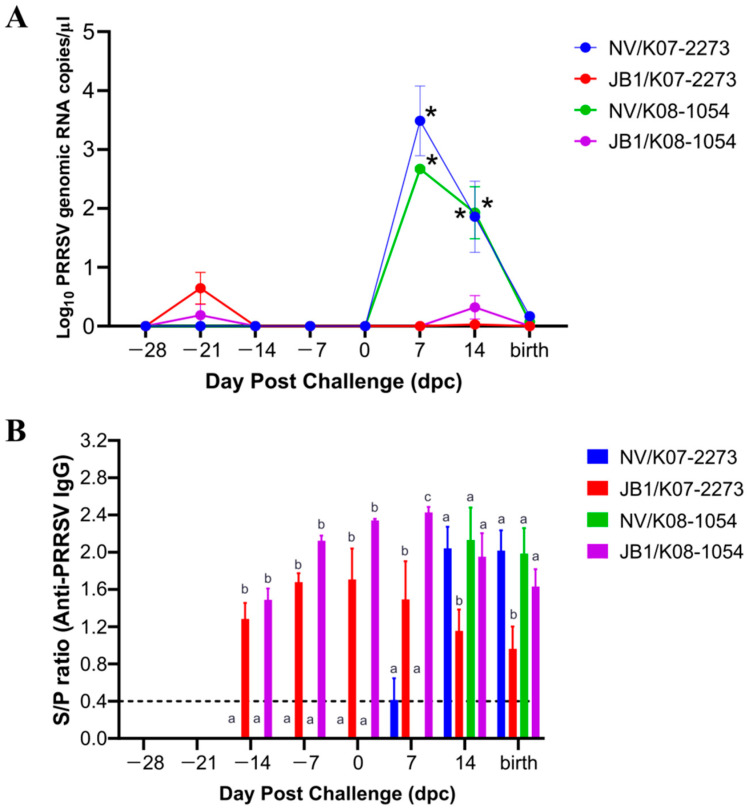
Mean values of the genomic copy number of PRRSV RNA and antibody response in the sera of pregnant sows from each group. (**A**) The genomic copy number of PRRSV RNA from pregnant sows post-vaccination and post-challenge. Data are shown as the means ± standard error of the mean (SEM). Asterisks indicate significant differences between the NV/K07–2273 and JB1/K07–2273 groups or between the NV/K08–1054 and JB1/K08–1054 groups (* *p* < 0.0001). (**B**) PRRSV-specific IgG response of vaccinated and challenged pregnant sows. Data are shown as the means ± SEM. Different letters represent significant differences among the experimental groups (*p* ≤ 0.05, Tukey’s test, two-way ANOVA).

PRRSV RNA was not detected in the sera of the NV groups before challenge. The JB1-vaccinated groups showed a mean peak of 0.7 log_10_ RNA copies/µL at −21 dpc (7 dpv), which was decreased to undetectable at −14 dpc (14 dpv) and maintained up to 7 dpc. After challenge with K07–2273 or K08–1054, the NV/K07–2273 and NV/K08–1054 groups exhibited peaks of 3.49 and 2.67 log_10_ RNA copies/µL at 7 dpc and 1.86 and 1.93 log_10_ RNA copies/µL at 14 dpc, respectively, which were significantly (*p* < 0.0001) higher than those of the JB1-vaccinated groups (Figure 3A). The JB1/K07–2273 and JB1/K08–1054 groups displayed mean peaks of 0.029 and 0.320 log_10_ RNA copies/µL, respectively, at 14 dpc, which became undetectable at 24 dpc (farrowing date). Overall, the JB1-vaccinated groups exhibited low viral RNA concentrations (<1.0 log_10_ RNA copies/µL) before the virus challenge and showed a reduction in viral RNA concentrations in comparison with those of the NV groups after the virus challenge.

### 3.2. The Levels of PRRSV-Specific IgG in the Sera from Sows

The levels of induced IgG were evaluated in sows following JB1 vaccination and PRRSV infection. The JB1-vaccinated groups became seropositive at −14 dpc (14 dpv) and were maintained until the end of the experiment. The JB1/K07–2273 group exhibited a mean peak IgG level of 1.71 S/P ratio at 0 dpc, which gradually decreased. In the case of the JB1/K08–1054 group, a mean peak IgG level of 2.43 S/P ratio was detected at 7 dpc and decreased through the last day of the experiment. The NV/K07–2273 infection group exhibited seroconversion at 7 dpc, and the NV/K08–1054 infection group was seropositive at 14 dpc (Figure 3B). The NV/K07–2273 and NV/K08–1054 groups showed the highest mean peak IgG levels of 2.04 and 2.13 S/P ratio at 14 dpc, respectively, which was maintained throughout the study period.

### 3.3. Measurement of SVN Antibodies(Log_2_)

SVN antibody titers were not observed in sera before vaccination. JB1 induced SVN antibody titers (log_2_) of 1 to 4 and 0.5 to 2.5 at 28 dpv (0 dpc) against K07–2273 and K08–1054, respectively. After the virus challenge, the JB1/K07–2273 group had SVN titersof 2.5 to 5.5 against K07–2273, while the JB1/K08–1054 group had SVN titers of 2.5 to 4 against K07–2273 at 14 dpc. In addition, SVN titers of 0.5 to 2.5 were observed in the JB1/K07–2273 group against K08–1054, while SVN titers of 0 to 1 were observed in the JB1/K08–1054 group against K08–1054 at 14 dpc (Table 2).

### 3.4. Litter Outcomes

Sows J1 and J2 (JB1/K07–2273 group) farrowed 9 and 6 live neonates at 113 and 115 days of gestation, respectively. In the JB1/K08–1054 group, J3 farrowed 12 neonates, but 1 neonate was stillborn at 113 days of gestation, while J4 farrowed 13 live neonates at 114 days of gestation. In contrast, J5 farrowed 10 live neonates and 2 dead neonates at 115 days of gestation, and J6 farrowed 4 live neonates and 8 dead neonates at 112 days of gestation. In addition, J7 and J8 farrowed 1/9 and 12/3 (stillborn/live born) neonates at 112 days of gestation (Table 3). Comparing stillborn rates by group, the JB1/K07–2273 group showed a 0% death rate, the JB1/K08–1054 group exhibited a 4% death rate, while the NV/K07–2273 and NV/K08–1054 groups showed 41.67% and 52.00% death rates, respectively.

**Figure 4:**
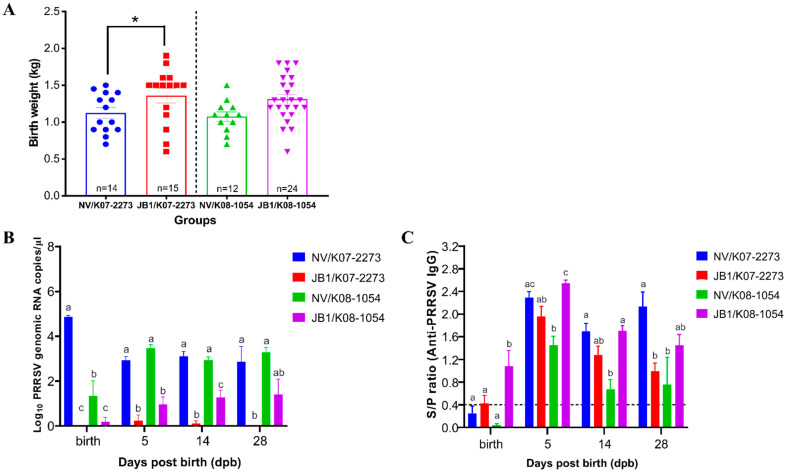
The birth weight of live-born piglets and results regarding the levels of PRRSV vertical transmission. (**A**) Mean birth weight values from live-born piglets of each group. Data are shown as the means ± standard error of the mean (SEM). Asterisks indicate significant differences between the NV/K07–2273 and JB1/K07–2273 groups or between the NV/K08–1054 and JB1/K08–1054 groups (* *p* < 0.05). (**B**) The genomic copy number of PRRSV RNA from piglets post-birth. Data are shown as the means ± SEM. Different letters represent significant differences among the experimental groups (*p* ≤ 0.05, Tukey’s test, two-way ANOVA). (**C**) PRRSV-specific IgG response of piglets. Data are shown as the means ± SEM. Different letters represent significant differences among the experimental groups (*p* ≤ 0.05, Tukey’s test, two-way ANOVA).

Precluding abnormal neonates, the birth weight of only live neonates was measured for accurate analysis. The JB1/K07–2273 group (*n* = 15) had an average birth weight of 1.36 ± 0.098 kg (mean ± SEM), which was significantly higher (*p* = 0.0252) than that of the NV/K07–2273 group (1.13 ± 0.072 kg) (*n* = 14). In addition, the birth weight of 1.313 ± 0.063 kg in the JB1/K08–1054 group (*n* = 24) was higher than that in the NV/K08–1054 group (*n* = 12, 1.08 ± 0.063 kg), although the difference was not statistically significant (Figure 4A).

### 3.5. The Levels of Viremia of Piglets

PRRSV RNA was observed at birth at concentrations of 4.87 and 1.33 log_10_ RNA copies/µL in the sera of the NV/K07–2273 and NV/K08–1054 group piglets, respectively. The piglets of the NV/K07–273 and NV/K08–1054 groups showed similar levels of approximately 3.0 log_10_ RNA copies/µL at 5 dpb, which were significantly higher (*p* < 0.001) than those of the JB1-vaccinated groups and were maintained throughout the experimental period. On the other hand, 0 and 0.19 log_10_ RNA copies/µL were detected in the sera of the JB1/K07–2273 and JB1/K08–1054 groups, respectively. In addition, the JB1/K07–2273 group had a significantly lower (*p* < 0.001) viral RNA concentration by 28 dpb than the NV groups. The JB1/K08–1054 group exhibited 0.97 log_10_ RNA copies/µL at 5 dpb, which was slightly increased to 1.41 log_10_ RNA copies/µL up to 28 dpb (Figure 4B).

### 3.6. PRRSV-Specific IgG of Piglets

The NV groups did not show significant PRRSV-specific IgG at birth. The JB1/K07–2273 and JB1/K08–1054 groups exhibited mean values of 0.43 and 1.09 S/P ratio at birth, respectively. The NV/K07–2273 and NV/K08–1054 groups showed mean peaks of 2.30 and 1.45 S/P ratio at 5 dpb, which were slightly decreased and maintained up to 28 dpb. In the case of the vaccinated sows, the JB1/K07–2273 and JB1/K08–1054 groups exhibited mean peaks of 1.96 and 2.54 S/P ratio, which gradually decreased and reached 0.99 and 1.45 S/P ratio at 28 dpb, respectively (Figure 4C).

### 3.7. Histopathological Evaluation

**Figure 5:**
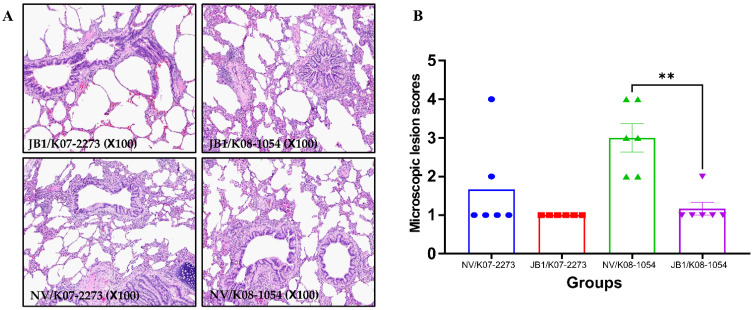
Histopathologic findings in the lungs of pregnant sows and piglets. (**A**) Representative pictures of lung lesions from sows. (**B**) Microscopic lesion scores of piglets (0, no lesion; 1, mild interstitial pneumonia; 2, moderate multifocal interstitial pneumonia; 3, moderate diffuse interstitial pneumonia; and 4, severe interstitial pneumonia). Data are shown as the means ± standard error of the mean (SEM). Asterisks indicate significant differences between the NV/K07–2273 and JB1/K07–2273 groups or between the NV/K08–1054 and JB1/K08–1054 groups (** *p* = 0.0065).

All sows were euthanized at 52 dpc, and the lung tissue of sows was subjected to histopathological examination. Minimal peribronchiolar and perivascular inflammatory cell infiltration was observed in JB1-vaccinated and NV sows. All piglets were also euthanized 28 days after birth, and the lung tissue of piglets was examined microscopically. Representative lesions of lungs from sows are shown in Figure 5A. The lung tissue of piglets from the JB1/K07–2273 and JB1/K08–1054 groups exhibited no remarkable lesions associated with PRRSV infection, but only one lung from a piglet from the JB1/K08–1054 group showed moderate multifocal interstitial pneumonia. The lung tissues from NV/K07–2273-infected piglets displayed mild interstitial pneumonia to severe interstitial pneumonia and were given a mean score of 1.67. In the case of lung lesions from the NV/K08–1054-infected group, the lung tissues showed moderate multifocal interstitial pneumonia to severe interstitial pneumonia and a mean score of 3.00, which was significantly higher (*p* = 0.0065) than that of the JB1/K08–1054 group (Figure 5B).

## 4. Discussion

It has been observed that PRRSV can cross the placenta and infect a few fetuses at an early stage of infection, and this process is dependent on the levels of viremia in sows [3,45,46]. For this reason, a reduction in viremia in the early stage of PRRSV infection is important for minimizing sow-to-piglet infection and is a useful indicator for evaluating vaccine efficacy [47,48]. In the current study, JB1-vaccinated sows showed a low PRRSV RNA concentration prior to the virus challenge and exhibited a significantly lower PRRSV RNA concentration after the virus challenge than NV sows. Although the viral RNA concentration of the JB1-vaccinated groups was low, the levels of anti-PRRSV IgG were sufficiently induced before the virus challenge. These results indicate that JB1 is safe and effectively reduces the viral concentration against two genetically different PRRSV strains.

PRRSV-specific VN antibodies are able to reduce the viremia, viral load in the lungs, and transplacental spread and protect against reproductive failure [49]. In the present study, JB1 induced mean SVN titers of over 1:8 against K07–2273 at 14 dpc in the JB1/K07–2273 and JB1/K08–1054 groups, while mean SVN titers of lower than 1:8 were observed against K08–1054. These results imply that the genomic composition of JB1, possessing ORFs 3–4 of K08–1054 and ORFs 5–6 of K07–2273, might induce different levels of SVN titers. Multiprotein complexes are formed by GP2, GP3, and GP4, which play a role in viral infectivity and receptor binding [50,51]. GP3 seems to be the main target of neutralizing antibodies from the blood of Lelystad (prototype of PRRSV-1)-infected pigs [52]. In addition, the GP3 chimeric PRRSV, which used the DNA shuffling method, induced neutralizing antibodies in pigs against a heterologous PRRSV strain [53]. Previous research demonstrated that Y^79^ and G^83^ in the nonoverlapping region of ORF3 (amino acid positions: 79–106), which is a B-cell epitope, played a critical role in the affinity of monoclonal antibodies [54]. In contrast, a previous study suggested that GP4 did not have an effect on PRRSV2 neutralization; if there was neutralization ability, it would be due to the impact of the overlapping region of GP3 and GP4 [18]. In contrast to GP3 and GP4, GP5 is a major glycosylated envelope protein that plays a role in the induction of VN antibody production [18,55]. The M protein is a non-glycosylated membrane protein that plays an important role in virus assembly and budding [56]. This protein forms heterodimers with GP5 by disulfide bonds, and GP5/M heterodimers are able to induce VN antibody production and lymphocyte proliferation [57,58,59,60]. For this reason, SVN titers of JB1-vaccinated groups against K08–1054 might be lower than those against K07–2273. Although lower SVN titers were induced by JB1 in pregnant sows, viremia in the JB1/K08–1054 group was significantly reduced in comparison with that in the NV/K08–1054 group. These results suggest that K08–1054-induced viremia was reduced in the JB1-vaccinated groups due to factors other than SVN titers. In a previous study [28], it was hypothesized that the reduction in viremia was caused by cytokines, such as TNF-α, IFN-γ, and IL-12, due to inoculation with CV, which has a more potent immune induction backbone (FL12). TNF-α induces inflammatory responses and inhibits PRRSV replication [61]. IFN-γ, which is an important cytokine associated with the cell-mediated immune (CMI) response, inhibits PRRSV replication [62,63]. In addition, IL-12 stimulates the differentiation of T cells and the production of IFN-γ and TNF-α [64,65,66]. Overall, JB1 may cross-protect against various PRRSV strains in pregnant sows.

Litter outcomes are an important parameter to evaluate vaccine efficacy in pregnant sows [47]. In the current study, vaccination of pregnant sows with JB1 followed by field isolate challenge exhibited improved fetal viability and piglet birth weight. A previous study found that pigs with low birth weight showed higher mortality prior to weaning and during the nursery phase. In addition, decreased birth weight resulted in inferior quality at weaning, finisher placement, and near the conclusion of finishing [67]. Therefore, the higher birth weight in the vaccinated groups than in nonvaccinated groups should be considered an important beneficial result.Furthermore, virological and serological assays were conducted on piglets over 28 days after birth to evaluate the levels of PRRSV vertical transmission. Piglets from the NV/K07–2273 and NV/K08–1054 groups showed significantly higher viremia than those from the JB1-vaccinated groups at birth, indicating that JB1 is able to reduce the viral concentration when PRRSV is transmitted across the placenta. However, the viral RNA concentration of sera from piglets in the JB1/K08–1054 group increased at 5 dpb, which continuously increased and reached a mean viral concentration of 1.41 log_10_ RNA copies/µL at 28 dpb. Sera with increased PRRSV RNA concentrations were subjected to ORF5 sequencing, and it was confirmed that the increased viral concentration was due to the K08–1054 strain (data not shown). In contrast to the JB1/K08–1054 group, the piglets of the JB1/K07–2273 group exhibited significantly fewer PRRSV genomic RNA copies during the experimental period. These results indicated that JB1 can completely prevent vertical transmission of K07–2273 but not K08–1054. This phenomenon might be associated with SVN titers or other factors, such as CMI. Nevertheless, considering that sows were challenged with viruses at 10^5^ TCID_50_/mL, which is a very high challenge dose that does not generally occur in the field, JB1 can significantly reduce virus transmission from sows to piglets.

Other previous studies suggested that histopathological lesion scores are also an important parameter of protection status and that this score has a correlation with the viral load in sera [68,69]. Similarly, in the present study, the piglets of the JB1-vaccinated groups did not show remarkable lesions related to PRRSV infection, while mild interstitial pneumonia to severe interstitial pneumonia was observed in the NV/K07–2273 and NV/K08–1054 group piglets. These results suggest that JB1 reduced viral replication and decreased the occurrence of lung lesions in piglets, indicating that JB1 provided simultaneous protection against both of the challenge viruses. In the case of sows, there was no difference in lung lesions between the JB1-vaccinated and NV groups. We speculate that the sows recovered from PRRSV infection because they were euthanized at 52 dpc.

## 5. Conclusions

To the best of our knowledge, this is the first study to evaluate the safety and efficacy of a chimeric vaccine in pregnant sows, including the assessment of viral vertical transmission from sows to piglets. In summary, pregnant sows in JB1-vaccinated groups exhibited reduced viremia against challenges with two genetically distinct PRRSV2 viruses, which induced higher levels of SVN titers in comparison with non-vaccinated sows. In addition, the JB1-vaccinated groups displayed improved piglet viability and birth weight. In the case of piglets from the sows of each group, JB1-vaccinated groups showed lower viremia and a more decreased degree of lung lesions compared with nonvaccinated groups.These results suggest that JB1 is an effective vaccine candidate and open new possibilities for cross-protection against various PRRSV strains. Furthermore, JB1 may be clinically effective in controlling reproductive failure, and JB1-based strategies can help to control PRRSV, which is prevalent in every country.

## Figures and Tables

**Table 1:** Shuttle vector *s*RVRp22_1a_ (*s*RVRp22_1a1_ + *s*RVRp22_1a2_) construction primers.

Primer Names	Sequences (5′–3′)	Reference	Constructed Name
F251-***SphI/BstZ17I***	***GCA TGC GCA TGC***GGA GGG CCA ***AGT ATA***CTG CAC ACG A	[41]	*s*RVRp22_1a1_
R4774-***SpeI***	***ACT AGT ACT AGT***GTG TCA GGG TCA ACC ACG A		
F4333-***SphI***	***GCA TGC GCA TGC***ATC TTG GCT GGA GCT TAC GT	[41]	*s*RVRp22_1a2_
R7821-***SpeI***	***ACT AGT ACT AGT***TGG TTG TGC TCA ACC GCG T		

Bold faced italic letters represent the restriction enzyme sequences.

**Table 2:** Measurement of serum virus-neutralizing antibody levels after vaccination and virus challenge.

Sow No.	Vaccinated	SVN Titer (log_2_) against K07–2273 (KorC)		SVN Titer (log_2_) against K08–1054 (L5)		Virus Challenged	SVN Titer (log_2_) against K07–2273 (KorC)	SVN Titer (log_2_) against K08–1054 (L5)
		−28 dpc	28 dpv (0 dpc)	−28 dpc	28 dpv (0 dpc)		14 dpc	14 dpc
J1	JB1	0	1	0	1	K07–2273	2.5	0.5
J2		0	4	0	2.5		5.5	2.5
J3		0	3	0	1	K08–1054	4	1
J4		0	2.5	0	0.5		2.5	0
J5	-	0	0	0	0	K07–2273	0	0
J6		0	0	0	0		0	0
J7		0	0	0	0	K08–1054	0	0
J8		0	0	0	0		0	0

**Table 3:** Summary of the reproductive evaluation results.

Sow No.	Vaccination	Infection	Day of Farrowing	*nd*^a^/*nb*^b^	Death Rate
J1	JB1	K07–2273	113	0/9	0.00%
J2			115	0/6	
J3		K08–1054	113	1/12	4.00%
J4			114	0/13	
J5	-	K07–2273	115	2/12	41.67%
J6			112	8/12	
J7		K08–1054	112	1/10	52.00%
J8			112	12/15	

^a^*nd*: The number of stillborn piglets. ^b^*nb*: The number of total born piglets.

## Data Availability

The datasets generated or analyzed during this study are available from the corresponding author on reasonable request.

## References

[1] Holtkamp D.J., Kliebenstein J.B., Neumann E.J., Zimmerman J.J., Rotto H.F., Yoder T.K., Wang C., Yeske P.E., Mowrer C.L., Haley C.A. (2013). Assessment of the economic impact of porcine reproductive and respiratory syndrome virus on United States pork producers. J. Swine Health Prod..

[2] Lunney J.K., Fang Y., Ladinig A., Chen N.H., Li Y.H., Rowland B., Renukaradhya G.J. (2016). Porcine Reproductive and Respiratory Syndrome Virus (PRRSV): Pathogenesis and interaction with the immune system. Annu. Rev. Anim. Biosci..

[3] Ladinig A., Detmer S.E., Clarke K., Ashley C., Rowland R.R., Lunney J.K., Harding J.C. (2015). Pathogenicity of three type 2 porcine reproductive and respiratory syndrome virus strains in experimentally inoculated pregnant gilts. Virus Res..

[4] Jeong J., Kim S., Park K.H., Kang I., Park S.J., Park C., Chae C. (2017). Evaluation of the effect of a porcine reproductive and respiratory syndrome (PRRS) modified-live virus vaccine on sow reproductive performance in endemic PRRS farms. Vet. Microbiol..

[5] Christianson W.T., Choi C.S., Collins J.E., Molitor T.W., Morrison R.B., Joo H.S. (1993). Pathogenesis of porcine reproductive and respiratory syndrome virus infection in mid-gestation sows and fetuses. Can. J. Vet. Res..

[6] Cavanagh D. (1997). Nidovirales: A new order comprising Coronaviridae and Arteriviridae. Arch. Virol..

[7] Meulenberg J.J., Hulst M.M., de Meijer E.J., Moonen P.L., den Besten A., de Kluyver E.P., Wensvoort G., Moormann R.J. (1993). Lelystad virus, the causative agent of porcine epidemic abortion and respiratory syndrome (PEARS), is related to LDV and EAV. Virology.

[8] Ruedas-Torres I., Rodriguez-Gomez I.M., Sanchez-Carvajal J.M., Larenas-Munoz F., Pallares F.J., Carrasco L., Gomez-Laguna J. (2021). The jigsaw of PRRSV virulence. Vet. Microbiol..

[9] Brinton M.A., Gulyaeva A.A., Balasuriya U.B.R., Dunowska M., Faaberg K.S., Goldberg T., Leung F.C.C., Nauwynck H.J., Snijder E.J., Stadejek T. (2021). ICTV virus taxonomy profile: Arteriviridae 2021. J. Gen. Virol..

[10] Han M., Yoo D. (2014). Engineering the PRRS virus genome: Updates and perspectives. Vet. Microbiol..

[11] Fang Y., Snijder E.J. (2010). The PRRSV replicase: Exploring the multifunctionality of an intriguing set of nonstructural proteins. Virus Res..

[12] Wissink E.H., Kroese M.V., van Wijk H.A., Rijsewijk F.A., Meulenberg J.J., Rottier P.J. (2005). Envelope protein requirements for the assembly of infectious virions of porcine reproductive and respiratory syndrome virus. J. Virol..

[13] Gonin P., Pirzadeh B., Gagnon C.A., Dea S. (1999). Seroneutralization of porcine reproductive and respiratory syndrome virus correlates with antibody response to the GP5 major envelope glycoprotein. J. Vet. Diagn. Investig..

[14] Plagemann P.G.W., Rowland R.R.R., Faaberg K.S. (2002). The primary neutralization epitope of porcine respiratory and reproductive syndrome virus strain VR-2332 is located in the middle of the GP5 ectodomain. Arch. Virol..

[15] Wissink E.H.J., van Wijk H.A.R., Kroese M.V., Weiland E., Meulenberg J.J.M., Rottier P.J.M., van Rijn P.A. (2003). The major envelope protein, GP5, of a European porcine reproductive and respiratory syndrome virus contains a neutralization epitope in its N-terminal ectodomain. J. Gen. Virol..

[16] Sun D., Khatun A., Kim W.I., Cooper V., Cho Y.I., Wang C., Choi E.J., Yoon K.J. (2016). Attempts to enhance cross-protection against porcine reproductive and respiratory syndrome viruses using chimeric viruses containing structural genes from two antigenically distinct strains. Vaccine.

[17] Cancel-Tirado S.M., Evans R.B., Yoon K.J. (2004). Monoclonal antibody analysis of porcine reproductive and respiratory syndrome virus epitopes associated with antibody-dependent enhancement and neutralization of virus infection. Vet. Immunol. Immunopathol..

[18] Kim W.I., Yoon K.J. (2008). Molecular assessment of the role of envelope-associated structural proteins in cross neutralization among different PRRS viruses. Virus Genes..

[19] Cao Q.M., Ni Y.Y., Cao D., Tian D., Yugo D.M., Heffron C.L., Overend C., Subramaniam S., Rogers A.J., Catanzaro N. (2018). Recombinant porcine reproductive and respiratory syndrome virus expressing membrane-bound interleukin-15 as an immunomodulatory adjuvant enhances NK and gammadelta T cell responses and confers heterologous protection. J. Virol..

[20] Shi M., Lam T.T.Y., Hon C.C., Hui R.K.H., Faaberg K.S., Wennblom T., Murtaugh M.P., Stadejek T., Leung F.C.C. (2010). Molecular epidemiology of PRRSV: A phylogenetic perspective. Virus Res..

[21] Lee J.A., Lee N.H., Lee J.B., Park S.Y., Song C.S., Choi I.S., Lee S.W. (2016). Genetic diversity of the Korean field strains of porcine reproductive and respiratory syndrome virus. Infect. Genet. Evol..

[22] Kang H., Yu J.E., Shin J.E., Kang A., Kim W.I., Lee C., Lee J., Cho I.S., Choe S.E., Cha S.H. (2018). Geographic distribution and molecular analysis of porcine reproductive and respiratory syndrome viruses circulating in swine farms in the Republic of Korea between 2013 and 2016. BMC Vet. Res..

[23] Kim H.K., Nguyen V.G., Kim I.O., Park J.H., Park S.J., Rho S.M., Han J.Y., Park B.K. (2012). Epidemiologic and phylogenetic characteristics of porcine reproductive and respiratory syndrome viruses in conventional swine farms of Jeju Island as a candidate region for PRRSV eradication. Transbound. Emerg. Dis..

[24] Khatun A., Shabir N., Yoon K.J., Kim W.I. (2015). Effects of ribavirin on the replication and genetic stability of porcine reproductive and respiratory syndrome virus. BMC Vet. Res..

[25] Charerntantanakul W. (2012). Porcine reproductive and respiratory syndrome virus vaccines: Immunogenicity, efficacy and safety aspects. World J. Virol..

[26] Murtaugh M.P., Xiao Z., Zuckermann F. (2002). Immunological responses of swine to porcine reproductive and respiratory syndrome virus infection. Viral. Immunol..

[27] Okuda Y., Kuroda M., Ono M., Chikata S., Shibata I. (2008). Efficacy of vaccination with porcine reproductive and respiratory syndrome virus following challenges with field isolates in Japan. J. Vet. Med. Sci..

[28] Shabir N., Khatun A., Nazki S., Kim B., Choi E.J., Sun D., Yoon K.J., Kim W.I. (2016). Evaluation of the cross-protective efficacy of a chimeric porcine reproductive and respiratory syndrome virus constructed based on two field strains. Viruses.

[29] Kim W.I., Kim J.J., Cha S.H., Yoon K.J. (2008). Different biological characteristics of wild-type porcine reproductive and respiratory syndrome viruses and vaccine viruses and identification of the corresponding genetic determinants. J. Clin. Microbiol..

[30] Nielsen H.S., Oleksiewicz M.B., Forsberg R., Stadejek T., Botner A., Storgaard T. (2001). Reversion of a live porcine reproductive and respiratory syndrome virus vaccine investigated by parallel mutations. J. Gen. Virol..

[31] Opriessnig T., Halbur P.G., Yoon K.J., Pogranichniy R.M., Harmon K.M., Evans R., Key K.F., Pallares F.J., Thomas P., Meng X.J. (2002). Comparison of molecular and biological characteristics of a modified live porcine reproductive and respiratory syndrome virus (PRRSV) vaccine (ingelvac PRRS MLV), the parent strain of the vaccine (ATCC VR2332), ATCC VR2385, and two recent field isolates of PRRSV. J. Virol..

[32] Lee J.A., Lee N.H., Lee S.W., Park S.Y., Song C.S., Choi I.S., Lee J.B. (2014). Development of a chimeric strain of porcine reproductive and respiratory syndrome virus with an infectious clone and a Korean dominant field strain. J. Microbiol..

[33] Choi H.-Y., Lee S.-H., Ahn S.-H., Choi J.-C., Jeong J.-Y., Lee B.-J., Kang Y.-L., Hwang S.-S., Lee J.-K., Lee S.-W. (2021). A chimeric porcine reproductive and respiratory syndrome virus (PRRSV)-2 vaccine is safe under international guidelines and effective both in experimental and field conditions. Res. Vet. Sci..

[34] Tian D., Cao D., Lynn Heffron C., Yugo D.M., Rogers A.J., Overend C., Matzinger S.R., Subramaniam S., Opriessnig T., LeRoith T. (2017). Enhancing heterologous protection in pigs vaccinated with chimeric porcine reproductive and respiratory syndrome virus containing the full-length sequences of shuffled structural genes of multiple heterologous strains. Vaccine.

[35] Levi L.I., Gnadig N.F., Beaucourt S., McPherson M.J., Baron B., Arnold J.J., Vignuzzi M. (2010). Fidelity variants of RNA dependent RNA polymerases uncover an indirect, mutagenic activity of amiloride compounds. PLoS Pathog..

[36] Feigelstock D.A., Mihalik K.B., Feinstone S.M. (2011). Selection of hepatitis C virus resistant to ribavirin. Virol. J..

[37] Pfeiffer J.K., Kirkegaard K. (2003). A single mutation in poliovirus RNA-dependent RNA polymerase confers resistance to mutagenic nucleotide analogs via increased fidelity. Proc. Natl. Acad. Sci. USA.

[38] Sierra M., Airaksinen A., Gonzalez-Lopez C., Agudo R., Arias A., Domingo E. (2007). Foot-and-mouth disease virus mutant with decreased sensitivity to ribavirin: Implications for error catastrophe. J. Virol..

[39] Khatun A., Shabir N., Seo B.J., Kim B.S., Yoon K.J., Kim W.I. (2016). The attenuation phenotype of a ribavirin-resistant porcine reproductive and respiratory syndrome virus is maintained during sequential passages in pigs. J. Virol..

[40] Miyazaki K., Takenouchi M. (2002). Creating random mutagenesis libraries using megaprimer PCR of whole plasmid. Biotechniques.

[41] Miyazaki K. (2011). MEGAWHOP cloning: A method of creating random mutagenesis libraries via megaprimer PCR of whole plasmids. Methods Enzym..

[42] Nielsen H.S., Liu G., Nielsen J., Oleksiewicz M.B., Botner A., Storgaard T., Faaberg K.S. (2003). Generation of an infectious clone of VR-2332, a highly virulent North American-type isolate of porcine reproductive and respiratory syndrome virus. J. Virol..

[43] Truong H.M., Lu Z., Kutish G.F., Galeota J., Osorio F.A., Pattnaik A.K. (2004). A highly pathogenic porcine reproductive and respiratory syndrome virus generated from an infectious cDNA clone retains the in vivo virulence and transmissibility properties of the parental virus. Virology.

[44] Yang M.-S., Jeong C.-G., Nazki S., Lee S.-M., Kim W.-I., Kim B. (2019). Comparison of immune cell populations in bronchoalveolar lavage cells and PBMC cytokine expressions in porcine reproductive and respiratory syndrome and porcine respiratory disease complex. Korean J. Vet. Serv..

[45] Ladinig A., Ashley C., Detmer S.E., Wilkinson J.M., Lunney J.K., Plastow G., Harding J.C. (2015). Maternal and fetal predictors of fetal viral load and death in third trimester, type 2 porcine reproductive and respiratory syndrome virus infected pregnant gilts. Vet. Res..

[46] Harding J.C.S., Ladinig A., Novakovic P., Detmer S.E., Wilkinson J.M., Yang T.F., Lunney J.K., Plastow G.S. (2017). Novel insights into host responses and reproductive pathophysiology of porcine reproductive and respiratory syndrome caused by PRRSV-2. Vet. Microbiol..

[47] Yang S., Oh T., Cho H., Chae C. (2020). A comparison of commercial modified-live PRRSV-1 and PRRSV-2 vaccines against a dual heterologous PRRSV-1 and PRRSV-2 challenge in late term pregnancy gilts. Comp. Immunol. Microb..

[48] Jeong J., Kim S., Park C., Park K.H., Kang I., Park S.J., Chae C. (2018). Commercial porcine reproductive and respiratory syndrome virus (PRRSV)-2 modified live virus vaccine against heterologous single and dual Korean PRRSV-1 and PRRSV-2 challenge. Vet. Rec..

[49] Vanhee M., Delputte P.L., Delrue I., Geldhof M.F., Nauwynck H.J. (2009). Development of an experimental inactivated PRRSV vaccine that induces virus-neutralizing antibodies. Vet. Res..

[50] Lee C.H., Bachand A., Murtaugh M.P., Yoo D.W. (2004). Differential host cell gene expression regulated by the porcine reproductive and respiratory syndrome virus GP4 and GP5 glycoproteins. Vet. Immunol. Immunopathol..

[51] Das P.B., Vu H.L.X., Dinh P.X., Cooney J.L., Kwon B., Osorio F.A., Pattnaik A.K. (2011). Glycosylation of minor envelope glycoproteins of porcine reproductive and respiratory syndrome virus in infectious virus recovery, receptor interaction, and immune response. Virology.

[52] Vanhee M., Van Breedam W., Costers S., Geldhof M., Noppe Y., Nauwynck H. (2011). Characterization of antigenic regions in the porcine reproductive and respiratory syndrome virus by the use of peptide-specific serum antibodies. Vaccine.

[53] Zhou L., Ni Y.Y., Pineyro P., Sanford B.J., Cossaboom C.M., Dryman B.A., Huang Y.W., Cao D.J., Meng X.J. (2012). DNA shuffling of the GP3 genes of porcine reproductive and respiratory syndrome virus (PRRSV) produces a chimeric virus with an improved cross-neutralizing ability against a heterologous PRRSV strain. Virology.

[54] Zhou Y.J., An T.Q., He Y.X., Liu J.X., Qiu H.J., Wang Y.F., Tong G. (2006). Antigenic structure analysis of glycosylated protein 3 of porcine reproductive and respiratory syndrome virus. Virus Res..

[55] Kim W.I., Kim J.J., Cha S.H., Wu W.H., Cooper V., Evans R., Choi E.J., Yoon K.J. (2013). Significance of genetic variation of PRRSV ORF5 in virus neutralization and molecular determinants corresponding to cross neutralization among PRRS viruses. Vet. Microbiol..

[56] Conzelmann K.K., Visser N., Van Woensel P., Thiel H.J. (1993). Molecular characterization of porcine reproductive and respiratory syndrome virus, a member of the arterivirus group. Virology.

[57] Wieringa R., de Vries A.A., van der Meulen J., Godeke G.J., Onderwater J.J., van Tol H., Koerten H.K., Mommaas A.M., Snijder E.J., Rottier P.J. (2004). Structural protein requirements in equine arteritis virus assembly. J. Virol..

[58] Nan Y., Wu C., Gu G., Sun W., Zhang Y.J., Zhou E.M. (2017). Improved vaccine against PRRSV: Current progress and future perspective. Front. Microbiol..

[59] Jiang Y., Xiao S., Fang L., Yu X., Song Y., Niu C., Chen H. (2006). DNA vaccines co-expressing GP5 and M proteins of porcine reproductive and respiratory syndrome virus (PRRSV) display enhanced immunogenicity. Vaccine.

[60] Jiang W.M., Jiang P., Li Y.F., Tang J.Y., Wang X.W., Ma S. (2006). Recombinant adenovirus expressing GP5 and M fusion proteins of porcine reproductive and respiratory syndrome virus induce both humoral and cell-mediated immune responses in mice. Vet. Immunol. Immunopathol..

[61] Lopez-Fuertes L., Campos E., Domenech N., Ezquerra A., Castro J., Domínguez J., Alonso F. (2000). Porcine reproductive and respiratory syndrome (PRRS) virus down-modulates TNF-α production in infected macrophages. Virus Res..

[62] Bautista E., Molitor T. (1999). IFNγ inhibits porcine reproductive and respiratory syndrome virus replication in macrophages. Arch. Virol..

[63] Li X., Galliher-Beckley A., Pappan L., Trible B., Kerrigan M., Beck A., Hesse R., Blecha F., Nietfeld J.C., Rowland R.R. (2014). Comparison of host immune responses to homologous and heterologous type II porcine reproductive and respiratory syndrome virus (PRRSV) challenge in vaccinated and unvaccinated pigs. Biomed. Res. Int..

[64] Dwivedi V., Manickam C., Binjawadagi B., Linhares D., Murtaugh M.P., Renukaradhya G.J. (2012). Evaluation of immune responses to porcine reproductive and respiratory syndrome virus in pigs during early stage of infection under farm conditions. Virol. J..

[65] Chung H.K., Chae C. (2003). Expression of interleukin-10 and interleukin-12 in piglets experimentally infected with porcine reproductive and respiratory syndrome virus (PRRSV). J. Comp. Pathol..

[66] Barranco I., Gomez-Laguna J., Rodriguez-Gomez I.M., Quereda J.J., Salguero F.J., Pallares F.J., Carrasco L. (2012). Immunohistochemical expression of IL-12, IL-10, IFN-alpha and IFN-gamma in lymphoid organs of porcine reproductive and respiratory syndrome virus-infected pigs. Vet. Immunol. Immunopathol..

[67] Fix J., Cassady J., Holl J., Herring W., Culbertson M., See M.J.L.S. (2010). Effect of piglet birth weight on survival and quality of commercial market swine. Livest. Sci..

[68] Han K., Seo H.W., Oh Y., Kang I., Park C., Chae C. (2013). Comparison of the virulence of European and North American genotypes of porcine reproductive and respiratory syndrome virus in experimentally infected pigs. Vet. J..

[69] Park C., Choi K., Jeong J., Chae C. (2015). Cross-protection of a new type 2 porcine reproductive and respiratory syndrome virus (PRRSV) modified live vaccine (Fostera PRRS) against heterologous type 1 PRRSV challenge in growing pigs. Vet. Microbiol..

